# Accelerating Research on Brain Aging: Enabling Brain Imaging Data Sharing in the Open Science Landscape

**DOI:** 10.1111/ene.70672

**Published:** 2026-07-24

**Authors:** Kathrin Giehl, Thilo van Eimeren

**Affiliations:** ^1^ University of Cologne Faculty of Medicine and University Hospital Cologne, Department of Nuclear Medicine Cologne Germany; ^2^ Research Centre Juelich Institute for Neuroscience and Medicine (INM‐II) Juelich Germany; ^3^ University of Cologne Faculty of Medicine and University Hospital Cologne, Department of Neurology Cologne Germany

**Keywords:** BIDS, brain health, European Health Data Space, GDPR, neuroimaging

## Abstract

The global burden of age‐associated diseases continues to grow. In particular, the accelerating impact of neurodegenerative diseases on individuals, communities and societies necessitates more effective approaches to diagnosis, prognosis and treatment of such disorders. Hence, the establishment of imaging biomarkers for early detection of disease, progression monitoring, and therapeutic evaluation is of utmost importance. Yet, despite the scientific consensus on the benefits of scientific collaboration and consequently medical innovation including biomarker development, substantial barriers for sharing neuroimaging data remain, demanding a transformation in how scientific data are generated, made accessible, re‐used and valued. These barriers range from technical and infrastructural limitations to legal and motivational challenges that hinder widespread adoption of open science practices. Here, we present a comprehensive overview of the current landscape of brain imaging data sharing in neurodegenerative disease research. We explore the status of preregistration, data harmonization and storage standardization, legal compliance, and researcher incentives. We highlight best practices before, during and after data generation and the pressing need for a coordinated strategy regarding simplified and unified legal frameworks compliant with the General Data Protection Regulation of the European Union. Finally, we advocate for the establishment of an academic credit system designed to reward data stewardship. Only with a combined effort from researchers, stakeholders and funding agencies including a sustained infrastructure investment and community education, the field can fully overcome inertia and move towards much‐desired open science, thereby fully leveraging shared data to improve patient outcomes and scientific discovery.

## 1 Introduction

Neurodegenerative diseases such as Alzheimer's and Parkinson's disease affect millions globally and are increasing in prevalence with aging populations [1, 2, 3, 4]. However, timely diagnosis, disease monitoring, and tracking the impact of novel, potentially disease‐modifying interventions remain elusive. Neuroimaging techniques, especially magnetic resonance imaging (MRI) and positron emission tomography (PET), have transformed our ability to visualize disease‐ and therapy‐related brain changes and derive biomarkers that support clinical and research objectives [5, 6]. Sharing of neuroimaging data between individual scientists and institutes on a national, international, and global level can accelerate the development of suitable imaging biomarkers and foster the translation of recent scientific advances into clinical practice, ultimately improving patient care [7]. Yet, the potential of neuroimaging research is often undermined by fragmented datasets, siloed research efforts, and barriers to data accessibility [8]. The open science movement seeks to address these challenges by promoting transparency, reproducibility, and the free exchange of research outputs, including raw neuroimaging data, offering several benefits [7, 8, 9].

### 1.1 Improved Reproducibility

Making raw imaging data and detailed preprocessing pipelines accessible enables independent research teams to replicate findings and verify results. This transparency helps reduce methodological variability and guards against unintentional biases that may arise from undisclosed data handling or analysis procedures. Moreover, reproducibility fosters scientific integrity and builds public trust in the reliability of neuroimaging findings, which is particularly crucial in clinical research contexts where results may influence diagnostic and therapeutic decisions.

### 1.2 Enhanced Machine Learning Performance

Machine learning and artificial intelligence (AI) applications in neuroimaging, such as (semi‐)automated diagnostics or disease progression modeling, demand vast and diverse labeled datasets to perform reliably. Data sharing allows researchers to compile large, heterogeneous training sets that improve model robustness and reduce overfitting, which is especially important when translating findings across populations and institutions. Greater generalizability makes these models more clinically useful and scalable, while shared benchmark datasets facilitate comparative evaluation and algorithm validation.

### 1.3 Meta‐Analytic Insights

Integrating data from multiple studies through meta‐analysis can reveal consistent patterns that might remain undetected in individual datasets due to limited sample sizes or demographic variance. Pooled analyses increase statistical power, enable more nuanced subgroup investigations, and reduce the risk of false positives.

### 1.4 Resource Efficiency

Reusing existing datasets minimizes redundant data collection efforts, conserves financial and human resources, and shortens the time between hypothesis generation and clinical application. Public investment in large‐scale imaging studies yields greater returns when data are accessible to a broader research community. Sharing also promotes interdisciplinary collaboration, allowing non‐imaging experts such as biostatisticians, computer scientists, and clinicians to engage with neuroimaging data and contribute new perspectives and methodologies.

Importantly, sharing brain imaging data in the Open Science framework is not merely a technical enhancement but a foundational shift in scientific culture. While it enables cross‐institutional collaboration, supports large‐scale AI applications, enhances statistical power, and democratizes access to research opportunities, the transformation firstly needs time, training, effort, and human as well as financial resources. Thus, systemic technical, legal, and motivational obstacles currently continue to hinder the full realization of its implementation and need to be overcome [8]. Here, we evaluate the current challenges within the field of neuroimaging and offer recommendations to promote sustainable data sharing practices in neurodegenerative disease research at every stage of the scientific circle.

## 2 Before Data Collection

### 2.1 Preregistration: Enabling Transparency From the Start

Preregistration involves documenting key aspects of a research project such as hypotheses, study design, and planned analyses prior to data collection. This Open Science practice is an essential component of research transparency, helping to clearly distinguish between confirmatory and exploratory research. It requires researchers to define their intentions in advance, thereby minimizing the risk of data dredging, p‐hacking, and HARKing (hypothesizing after results are known), which can compromise the integrity of scientific findings [10, 11]. Registered Reports go a step further by integrating preregistration into the peer review process [12]. In this model, journals review and provisionally accept study protocols before results are known, committing to publication as long as the proposed methodology is followed. This approach shifts the focus of evaluation from outcomes to methodological rigor, reducing publication bias and promoting the dissemination of null or negative results [13].

Despite broad conceptual support, actual uptake of preregistration within the neuroimaging community remains modest. Common barriers include time constraints, lack of familiarity with available platforms, and concerns about potential impacts on publication opportunities. Additionally, some researchers perceive preregistration as inflexible, fearing that necessary methodological changes may later be viewed unfavorably [8]. To address these concerns, a variety of user‐friendly platforms and templates have emerged to facilitate the preregistration process. AsPredicted.org and the Open Science Framework [14] provide structured, intuitive workflows including dedicated templates for fMRI studies. Researchers can also choose between public or embargoed preregistrations, allowing for strategic flexibility. Crucially, deviations from the registered plan can be made, transparently reported, and justified in final publications. Beyond improving reproducibility, preregistration offers practical benefits: it encourages thoughtful planning, promotes resource efficiency, and provides a defensible record of research integrity. Funders and journals may accelerate its adoption by incentivizing preregistration through targeted calls, dedicated publication tracks, and recognition in grant evaluations. In a competitive research environment, such institutional support is vital to help embed preregistration as a routine scientific norm.

### 2.2 Harmonization of Image Acquisition

Variability in scanner hardware, software versions, pulse sequences, and reconstruction algorithms complicates data harmonization across data acquisition sites. Therefore, redefining and sharing acquisition parameters and pipelines is crucial for multicentre studies and the reuse of neuroimaging data by collaborating research groups. Currently, full acquisition harmonization remains aspirational, but sharing acquisition and processing metrics accompanied by raw images offers the possibility to mitigate some variability within the data. Aiming to overcome some of the potential obstacles for sharing of acquisition parameters and processing protocols, initiatives such as the Quantitative Imaging Biomarkers Alliance (QIBA) and Harmonizing Brain Imaging Methods for Vascular Contributions to Neurodegeneration (HARNESS) offer acquisition protocols, rating scales, and analysis templates [15, 16].

## 3 During Data Collection

### 3.1 Data Standardization and Storage

Another primary obstacle of data sharing in neuroimaging research is the lack of standardization in how neuroimaging data are structured, named, and annotated (i.e., “data‐level harmonization”) as well as processed and analyzed (i.e., “analysis‐level harmonization”).

Regarding data‐level harmonization, diverse directory formats, inconsistent file naming conventions, and insufficient metadata documentation impede the interoperability of datasets between institutions and software tools. This heterogeneity not only complicates collaboration, but also increases the risk of errors during data processing and interpretation. The Brain Imaging Data Structure (BIDS) addresses these issues by providing a community‐driven standard for organizing and describing neuroimaging data in a consistent, machine‐readable format [17, 18]. Originally designed for MRI data, BIDS has expanded to encompass a wide range of modalities, including PET [19], Magnetoencephalography [20] and Electroencephalography [21]. Its modular architecture facilitates the addition of new imaging types, supporting evolving research needs. BIDS simplifies the sharing and reuse of datasets by ensuring compatibility with widely used neuroimaging software packages, such as Statistical Parametric Mapping, the FMRIB Software Library and FreeSurfer. BIDS‐compliant datasets can be readily processed through standardized, automated workflows using BIDS Apps, which encapsulate entire analysis pipelines [22]. This not only improves reproducibility but also reduces technical barriers for less experienced users. Despite widespread endorsement by the neuroimaging community, the adoption of BIDS in practice faces challenges [8]. Many research groups struggle with the initial conversion of legacy datasets, particularly when metadata are incomplete or inconsistently recorded. In addition, the conversion to BIDS format requires technical know‐how and, in some cases, customized scripts or third‐party tools. To support the transition, a number of community‐developed resources have emerged. These include GitHub‐hosted tools for DICOM‐to‐BIDS conversion (e.g., HeuDiConv) [23], visual BIDS validators [24], example datasets [25] and comprehensive documentation [26]. Online tutorials and community forums further facilitate user onboarding.

But sustainable BIDS implementation requires more than open‐source tools; it also depends on dedicated personnel and structured training and thus institutional support. Institutions and funding agencies should invest in data stewards and research software engineers who can assist in BIDS compliance and metadata curation [8]. Integrating BIDS training into graduate education and providing incentives for sharing BIDS‐formatted datasets will further drive adoption. In the long term, the widespread use of BIDS can transform neuroimaging research by promoting interoperability, enabling large‐scale analyses, and streamlining regulatory oversight. Combined with FAIR data principles [27, 28], BIDS offers a robust framework for achieving transparency, efficiency, and replicability in brain imaging studies.

Beyond data organization, analysis‐level harmonization refers to the standardization of preprocessing pipelines, analytical workflows, and data derivates across studies. Even when datasets adhere to common formats such as BIDS, variability in preprocessing steps (e.g., motion correction, normalization, smoothing), software tools, parameter settings, and versioning can lead to substantial differences in downstream results. To address this, harmonization efforts must include not only standardized data structures but also consistent, well‐documented, and ideally version‐controlled analysis workflows. The use of containerized pipelines, open‐source tools, and shared benchmarking datasets can improve reproducibility and comparability across studies. In addition, the sharing of well‐characterized data derivatives, along with detailed descriptions of the pipelines used to generate them, can facilitate reuse and clinical translation [8].

Several operational ecosystems already demonstrate how large‐scale neuroimaging data sharing can be successfully implemented. Platforms such as EBRAINS (ebrains.eu) provide integrated infrastructures for storing, accessing, and analyzing multimodal brain data across institutions, enabling cross‐border collaboration and reproducible workflows. Established consortia further highlight the value of structured governance and standardized protocols. The Alzheimer's Disease Neuroimaging Initiative (ADNI; adni.loni.usc.edu) and the Parkinson's Progression Markers Initiative (PPMI; ppmi‐info.org) have pioneered open, harmonized data‐sharing models that support biomarker discovery and translational research. Together, these initiatives show that effective data sharing relies not only on conceptual frameworks but also on robust governance and clear standards.

## 4 After Data Collection

### 4.1 Legal and Ethical Constraints

Protecting personal data, especially personal health data, is important. Within the European Union (EU), the EU General Data Protection Regulation (GDPR) sets the rules for personal data, including neuroimaging data [29]. However, implementation of the GDPR varies by country, creating uncertainty for researchers and their legal counselors. This variability affects data transfer agreements (DTAs), informed consent procedures, and Institutional Review Board approvals, ultimately delaying scientific collaboration and medical innovation including the development of new therapeutic strategies directly impacting patients' quality of life [30, 31].

Under GDPR, pseudonymized data is still considered personal data, while fully anonymized data falls outside the regulation's scope. Thus, fully anonymized data can be shared much easier than pseudonymized data. However, in the context of research, full anonymization remains challenging and often a matter of debate. Firstly, for researchers invested in the ongoing creation of novel data sets, full anonymization of data right after their collection is not attractive nor feasible. Often a link to the data donor (i.e., the participant or patient) has to be maintained at least until the data collection is completed and the data base locked. Moreover, especially in the field of neuroimaging research, instant full anonymization might be not in the best interest of the patient. When undergoing neuroimaging, incidental findings are always a possibility, making contacting the patient an ethical necessity.

Secondly, it is currently debated how and when full anonymization could be reached. This issue relates to the interpretation of risk for re‐identification within the GDPR, which is based on an estimation. Data can be considered anonymous if the effort for re‐identification is estimated unreasonable. However, what is considered unreasonable often varies depending on the data at hand and legal representatives involved; a classic point of discussion e.g., is whether pseudonymized data that has been transferred to a collaborator without a subject identification list and is subsequently re‐coded can be considered anonymized as the risk of double re‐identification appears relatively low. Taken together, the risk‐based standard of the GDPR for re‐identification complicates compliance and gives ample opportunity for interpretation resulting in lengthy discussions between legal representatives of different institutions or universities, often delaying data transfer.

Initiatives like the Open Brain Consent offer GDPR‐compliant templates for informed consent and DTAs [32]. These tools can reduce administrative burdens and promote consistent practices. Engaging institutional legal departments early in study planning facilitates smoother ethics approval. Still, many institutions lack the internal legal expertise required to interpret GDPR provisions in the neuroimaging research context effectively, and ethics board reviews are often prolonged. To streamline compliance, a harmonized EU‐level manual or guidance document tailored to neuroimaging research is urgently needed to clarify expectations and procedures. Direct engagement and close collaboration with institutional data protection officers and legal representatives, and the use of standardized templates are essential for mitigating this legal bottleneck [8].

An effective de‐identification strategy is crucial for balancing data utility and participant privacy. In neuroimaging, this process involves more than scrubbing identifiers from DICOM headers. Structural MRI images contain facial features that could potentially be reconstructed using deep learning [33], raising another level of privacy concerns. However, despite this theoretical risk, one should remember that to date there are no documented cases of malicious re‐identification from neuroimaging data [34]. Nevertheless, aiming to reduce re‐identification risk from structural neuroimaging data defacing algorithms can be applied to remove facial features from structural scans [35, 36]. However, these tools can degrade image quality and interfere with some analytic pipelines. Alternative approaches include skull‐stripping, synthetic face generation, and robust metadata handling. Regardless of the method, anonymization must be tailored to the dataset and documented clearly to satisfy both ethical and legal standards. Beyond individual practices, community‐wide adoption of de‐identification protocols and standard risk assessments will ensure consistency and build trust among data contributors, users, and oversight bodies. The European Health Data Space initiative presents an opportunity to develop centralized standards and encourage cross‐border cooperation on privacy‐preserving data sharing [37].

In terms of privacy and legal aspects, a key, yet often underemphasized, aspect of neuroimaging data sharing is also the choice between centralized and federated data sharing architectures. In centralized models, data are transferred to a single repository, enabling standardized processing and efficient large‐scale analyses. While widely used in major consortia, such approaches require extensive legal agreements and raise concerns regarding data sovereignty, security, and compliance, particularly under the GDPR. Federated architectures, in contrast, allow data to remain at local sites while enabling joint analyses through distributed computing. Instead of transferring data, algorithms are deployed locally, and only aggregated results are shared. This approach reduces privacy risks and facilitates compliance with data protection regulations, making it especially suitable for sensitive clinical datasets. Federated models can help overcome key GDPR‐related barriers, including restrictions on cross‐border data transfer, while allowing institutions to retain control over their data. However, they require robust infrastructure, harmonized data standards, and secure communication protocols.

Related to this issue, questions around ownership and intellectual property (IP) present a further challenge in data sharing. Unlike a publication, where authorship clearly conveys ownership, data are often the product of collaborative efforts involving researchers, institutions, and funding bodies. Consequently, determining data ownership can be ambiguous. In most cases, institutions where data are collected retain IP rights. However, public funding agencies increasingly view data generated with taxpayer support as a public good. As such, they advocate for open access, though often permit embargo periods to allow original investigators time to publish primary findings. Clear licensing terms and DTAs are essential. These should specify usage rights, sharing restrictions, and responsibilities for derivative data. Allowing for controlled access rather than open distribution can address both privacy and IP concerns. Including statements about expected data use and attribution in consent forms and DTAs will foster transparency and trust. Ultimately, harmonized licensing frameworks that protect data providers' interests while promoting access are needed. Community consensus around principles for shared ownership and proper attribution will support more equitable and sustainable data sharing practices.

### 4.2 Aligning Incentives With Open Science Calls for a Cultural Shift

Despite broad support for open science in principle, the current academic reward system is fundamentally misaligned with its demands, prioritizing publications over data sharing and thereby hindering practical implementation of open science practices [8]. Reducing scientific performance indicators to publications neglects the labour‐intensive tasks of data acquisition, curation, documentation, and dissemination. In this respect, junior researchers and research support staff are particularly disadvantaged, receiving little credit for their essential roles in data acquisition and preparation. Scientific careers are traditionally advanced through first‐author publications in high‐impact journals. However, creating and maintaining high‐quality datasets demands substantial time, technical expertise, and infrastructure. All of those may go unrecognized under current evaluation metrics. Moreover, fears of data misuse, loss of competitive advantage, or lack of appropriate citation discourage many from sharing. To address this issue, a new incentive model that treats data generation and curation as primary scholarly outputs is required [8]. Practically, this could be archived in three steps: In an initial step, such as system should link datasets with unique publication identifiers (e.g., digital object identifiers) and associate them with the data creator, e.g., via a persistent researcher identifier such as ORCIDs. In a second step, evaluation criteria of funders and universities need to be revised in order to recognize data sets as primary scholarly outputs, thereby incorporating data sharing metrics into grant reviews, funding decisions, hiring processes and academic promotions. In a final step, implementation of sharable recognition is needed. This could be enabled by tracking dataset reuse and citations in bibliometric databases as well as the development of citation metrics that reflect dataset impact, visibility, and reuse frequency.

Such a system would make data creation contributions visible, measurable, and actively add to a researcher's measurable academic impact while specifically enabling recognition of technical staff, coordinators, and junior researchers whose work underpins successful data‐driven science. Importantly, funding agencies and institutions play a central role in operationalizing these changes. Grant review processes could explicitly evaluate prior data sharing contributions, while institutions could recognize and reward the development of high‐impact datasets as a distinct form of scholarly achievement. This would create tangible incentives for researchers to invest in high‐quality data curation and sharing. While the Global Research Council and major funding agencies are increasingly recognizing the need for updated assessment frameworks [38, 39], these efforts currently remain to be translated into concrete policies to reward data sharing as a first‐class scholarly activity.

## 5 Conclusion

The sharing of brain imaging data is essential to medical advancement, e.g., in biomarker development for neurodegenerative diseases, and therefore the effective diagnosis and treatment of such conditions. Currently, systemic challenges remain; especially those relating to data standardization, data privacy, and incentives. However, the promise of neuroimaging to transform our understanding and treatment of neurodegenerative diseases hinges on collaborative, transparent, and equitable data sharing. Technical solutions exist, legal frameworks can be harmonized, and motivational incentives can be realigned, given a coordinated effort of researchers, institutions, funders, and policymakers (see Table 1). The next generation of neuroscientific discovery depends on the collective ability to make neuroimaging data not only accessible but also meaningful and rewarding to share. Institutional leadership, cross‐sector partnerships, and robust community engagement are critical to overcoming inertia and establishing open data as the norm rather than the exception.

**TABLE 1:** Policy recommendations and strategic priorities.

Support technical infrastructure and standardization	Expand funding for BIDS‐compatible repositories and tools Maintain open‐source software for data conversion and validation Encourage platforms that store and manage both raw and derivative imaging data
Harmonize GDPR implementation	Develop EU‐level guidance specific to neuroimaging Promote adoption of standard consent and DTA templates (e.g., Open Brain Consent) Support ethics boards, data protection officers and legal representatives with specialized training in biomedical data governance
Enhance researcher training and support	Integrate open science best practices into academic curricula Fund dedicated roles (e.g., data stewards) to support compliance and quality control Provide workshops and mentoring for implementing BIDS and FAIR principles
Realign academic incentives	Promote use of persistent identifiers and track dataset reuse Develop institutional policies that reward data transparency and collaboration Recognize dataset contributions as primary scholarly outputs in tenure, promotion, and funding decisions
Foster cultural change through leadership	Encourage consortia and funding bodies to set data sharing standards Celebrate examples of impactful shared data initiatives (e.g., Alzheimer's Disease Neuroimaging Initiate, Parkinson's Progression Marker Initiative) Build a community ethos that values collaboration, openness, and reproducibility

## Author Contributions

**Kathrin Giehl:** conceptualization, writing – original draft. **Thilo van Eimeren:** conceptualization, writing – review and editing, supervision.

## Conflicts of Interest

Kathrin Giehl received a speaker fee from Synaptikon GmbH. Thilo van Eimeren received honoraria, stipends, or speaker fees from the Lundbeck Foundation, Gain Therapeutics, Orion Pharma, Lundbeck Pharma, Atheneum, and the International Movement Disorders Society. He receives materials from Life Molecular Imaging and Lilly Pharma. He owns stocks of the corporations NVIDIA, Microsoft, and I.B.M.

## Data Availability

Data sharing not applicable to this article as no datasets were generated or analysed during the current study.

## References

[1] J. P. Bach , U. Ziegler , G. Deuschl , R. Dodel , and G. Doblhammer‐Reiter , “Projected Numbers of People With Movement Disorders in the Years 2030 and 2050,” Movement Disorders 26 (2011): 2286–2290. 22021158 10.1002/mds.23878

[2] G. Deuschl , E. Beghi , F. Fazekas , et al., “The Burden of Neurological Diseases in Europe: An Analysis for the Global Burden of Disease Study 2017,” Lancet Public Health 5 (2020): e551–e567. 33007212 10.1016/S2468-2667(20)30190-0

[3] E. R. Dorsey , A. Elbaz , E. Nichols , et al., “Global, Regional, and National Burden of Parkinson's Disease, 1990–2016: A Systematic Analysis for the Global Burden of Disease Study 2016,” Lancet Neurology 17 (2018): 939–953. 30287051 10.1016/S1474-4422(18)30295-3 PMC6191528

[4] C. P. Ferri , M. Prince , C. Brayne , et al., “Global Prevalence of Dementia: A Delphi Consensus Study,” Lancet 366 (2005): 2112–2117. 16360788 10.1016/S0140-6736(05)67889-0 PMC2850264

[5] O. Hansson , “Biomarkers for Neurodegenerative Diseases,” Nature Medicine 27 (2021): 954–963. 10.1038/s41591-021-01382-x 34083813

[6] C. G. Schwarz , “Uses of Human MR and PET Imaging in Research of Neurodegenerative Brain Diseases,” Neurotherapeutics 18 (2021): 661–672. 33723751 10.1007/s13311-021-01030-9 PMC8423895

[7] M. P. Milham , R. C. Craddock , J. J. Son , et al., “Assessment of the Impact of Shared Brain Imaging Data on the Scientific Literature,” Nature Communications 9 (2018): 2818. 10.1038/s41467-018-04976-1 PMC6053414 30026557

[8] K. Giehl , H.‐J. Mutsaerts , K. Aarts , et al., “Sharing Brain Imaging Data in the Open Science Era: How and Why?,” Lancet Digital Health 6 (2024): e526–e535. 38906618 10.1016/S2589-7500(24)00069-4

[9] ALLEA , “The European Code of Conduct for Research Integrity,” (2023), https://allea.org/wp‐content/uploads/2023/06/European‐Code‐of‐Conduct‐Revised‐Edition‐2023.pdf.

[10] N. L. Kerr , “HARKing: Hypothesizing After the Results Are Known,” Personality and Social Psychology Review 2 (1998): 196–217. 15647155 10.1207/s15327957pspr0203_4

[11] J. P. Simmons , L. D. Nelson , and U. Simonsohn , “False‐Positive Psychology: Undisclosed Flexibility in Data Collection and Analysis Allows Presenting Anything as Significant,” Psychological Science 22 (2011): 1359–1366. 22006061 10.1177/0956797611417632

[12] S. L. Stewart , E. M. Rinke , R. McGarrigle , et al., “Pre‐Registration and Registered Reports: A Primer From UKRN,” (2020).

[13] R. Rosenthal , “The File Drawer Problem and Tolerance for Null Results,” Psychological Bulletin 86 (1979): 638–641.

[14] “Open Science Framework (OSF),” (2012), https://osf.io.

[15] A. Shukla‐Dave , N. A. Obuchowski , T. L. Chenevert , et al., “Quantitative Imaging Biomarkers Alliance (QIBA) Recommendations for Improved Precision of DWI and DCE‐MRI Derived Biomarkers in Multicenter Oncology Trials,” Journal of Magnetic Resonance Imaging 49 (2019): e101–e121. 30451345 10.1002/jmri.26518 PMC6526078

[16] E. E. Smith , G. J. Biessels , F. De Guio , et al., “Harmonizing Brain Magnetic Resonance Imaging Methods for Vascular Contributions to Neurodegeneration,” Alzheimer's & Dementia: Diagnosis, Assessment & Disease Monitoring 11 (2019): 191–204. 10.1016/j.dadm.2019.01.002 PMC6396326 30859119

[17] R. A. Poldrack , C. J. Markiewicz , S. Appelhoff , et al., “The Past, Present, and Future of the Brain Imaging Data Structure (BIDS),” Imaging Neuroscience 2 (2024): 1–19. 10.1162/imag_a_00103 PMC11415029 39308505

[18] K. J. Gorgolewski , T. Auer , V. D. Calhoun , et al., “The Brain Imaging Data Structure, a Format for Organizing and Describing Outputs of Neuroimaging Experiments,” Scientific Data 3 (2016): 1–9. 10.1038/sdata.2016.44 PMC4978148 27326542

[19] M. Norgaard , G. J. Matheson , H. D. Hansen , et al., “PET‐BIDS, an Extension to the Brain Imaging Data Structure for Positron Emission Tomography,” Scientific Data 9 (2022): 65. 35236846 10.1038/s41597-022-01164-1 PMC8891322

[20] G. Niso , K. J. Gorgolewski , E. Bock , et al., “MEG‐BIDS, the Brain Imaging Data Structure Extended to Magnetoencephalography,” Scientific Data 5 (2018): 1–5. 29917016 10.1038/sdata.2018.110 PMC6007085

[21] C. R. Pernet , S. Appelhoff , K. J. Gorgolewski , et al., “EEG‐BIDS, an Extension to the Brain Imaging Data Structure for Electroencephalography,” Scientific Data 6 (2019): 103. 31239435 10.1038/s41597-019-0104-8 PMC6592877

[22] K. J. Gorgolewski , F. Alfaro‐Almagro , T. Auer , et al., “BIDS Apps: Improving Ease of Use, Accessibility, and Reproducibility of Neuroimaging Data Analysis Methods,” PLoS Computational Biology 13 (2017): e1005209. 28278228 10.1371/journal.pcbi.1005209 PMC5363996

[23] Y. O. Halchenko , M. Goncalves , S. Ghosh , et al., “HeuDiConv—Flexible DICOM Conversion Into Structured Directory Layouts,” Journal of Open Source Software 9 (2024): 5839. 39323511 10.21105/joss.05839 PMC11423922

[24] “BIDS Validator,” https://bids‐standard.github.io/bids‐validator/.

[25] “Bids‐Examples,” https://github.com/bids‐standard/bids‐examples.

[26] “The Brain Imaging Data Structure (BIDS) Specification – Introduction,” https://bids‐specification.readthedocs.io/en/stable/introduction.html.

[27] M. D. Wilkinson , M. Dumontier , I. J. Aalbersberg , et al., “The FAIR Guiding Principles for Scientific Data Management and Stewardship,” Scientific Data 3 (2016): 1–9. 10.1038/sdata.2016.18 PMC4792175 26978244

[28] M. D. Wilkinson , M. Dumontier , I. Jan Aalbersberg , et al., “Addendum: The FAIR Guiding Principles for Scientific Data Management and Stewardship,” Scientific Data 6 (2019): 6. 30890711 10.1038/s41597-019-0009-6 PMC6427092

[29] Union E , “Regulation (EU) 2016/679,” (2016).

[30] D. Peloquin , M. DiMaio , B. Bierer , and M. Barnes , “Disruptive and Avoidable: GDPR Challenges to Secondary Research Uses of Data,” European Journal of Human Genetics 28 (2020): 697–705. 32123329 10.1038/s41431-020-0596-x PMC7411058

[31] R. Eiss , “Confusion Over Europe's Data‐Protection Law Is Stalling Scientific Progress,” Nature 584 (2020): 498–499. 32843731 10.1038/d41586-020-02454-7

[32] E. Bannier , G. Barker , V. Borghesani , et al., The Open Brain Consent: Informing Research Participants and Obtaining Consent to Share Brain Imaging Data (Wiley Online Library, 2021), 1945–1951. 10.1002/hbm.25351 PMC8046140 33522661

[33] D. Eke , I. E. Aasebø , S. Akintoye , et al., “Pseudonymisation of Neuroimages and Data Protection: Increasing Access to Data While Retaining Scientific Utility,” Neuroimage: Reports 1 (2021): 100053. 40568426 10.1016/j.ynirp.2021.100053 PMC12172758

[34] A. Jwa , O. Koyejo , and R. Poldrack , “Demystifying the risk of reidentification in neuroimaging data–a technical and regulatory analysis,” (2023). 10.1162/imag_a_00111 PMC12247609 40800528

[35] C. G. Schwarz , W. K. Kremers , H. J. Wiste , et al., “Changing the Face of Neuroimaging Research: Comparing a New MRI de‐Facing Technique With Popular Alternatives,” NeuroImage 231 (2021): 117845. 33582276 10.1016/j.neuroimage.2021.117845 PMC8154695

[36] D. A. Clunie , A. Flanders , A. Taylor , et al., “Report of the Medical Image De‐Identification (MIDI) Task Group – Best Practices and Recommendations,” Arxiv (2025): 2303.10473 v10473.

[37] “European Health Data Space (EHDS) | Updates,” https://www.european‐health‐data‐space.com.

[38] H. H. Pierce , A. Dev , E. Statham , and B. E. Bierer , “Credit data generators for data reuse,” Nature 570 (2019): 30–32. 31164773 10.1038/d41586-019-01715-4

[39] “Statement of Principles on Recognising and Rewarding Researchers,” (2023), https://globalresearchcouncil.org/fileadmin//documents/GRC_Publications/SoP_Recognising_and_Rewarding_Researchers.pdf.

